# #BeSeen: understanding young people’s views of the motivation and impacts of sharing self-harm imagery online and use of their social media data for research—a UK participatory arts-led qualitative study

**DOI:** 10.1136/bmjopen-2023-076981

**Published:** 2024-07-23

**Authors:** Dana Dekel, Amanda Marchant, Todd Smith, Harley Morgan, Sarah Tombs, Ashrafunnesa Khanom, Karen Ingham, Ann John

**Affiliations:** 1 Medical School, Swansea University, Swansea, UK; 2 Public Health Wales NHS Trust, Cardiff, UK; 3 Independent Artist, Swansea, UK

**Keywords:** adolescent, mental health, qualitative research, suicide & self-harm

## Abstract

**Abstract:**

**Objectives:**

This study explored the views of young people from diverse backgrounds, with or without a history of self-harm, on the motivation and impacts of sharing self-harm imagery online and the use of their social media data for mental health research.

**Design:**

Thematic analysis of 27 semi-structured one-to-one interviews.

**Setting:**

Two workshops were conducted in 2021.

**Participants:**

We recruited 27 study participants aged 16–24 (60% male). Sixteen (59%) participants were refugee and asylum seekers (RAS).

**Results:**

Two main themes were generated: (1) *Online imagery of self-harm* captured perceived motivations for sharing such images, the potential impacts on others and possible need of censorship. This theme was characterised by mixed attitudes towards motivations for sharing, with some perceiving this as attention seeking, while others thought of it as help seeking or sharing of pain. Overall, participants agreed that images of self-harm can be triggering and should include trigger warnings. (2) *Data sharing for mental health and self-harm research* captured views on the use of social media posts and images for research purposes, and levels of trust in public and private organisations. It outlined positive views on their data being shared for research for public benefit, but highlighted issues of consent. The two most trusted organisations to hold and conduct research were the National Health Service and Universities. Participants from the RAS group were more inclined to agree to their data being used and had higher levels of trust in government.

**Conclusion:**

Young people care about their privacy and use of their data even when it is publicly available. Coproduction with young people of resources to support understanding and develop innovative solutions to gaining informed consent for data sharing and research for public benefit is required. Young people from excluded communities, post-immigration RAS and males should be purposively involved in future social media research.

**STRENGTHS AND LIMITATIONS OF THIS STUY**

- This is the first study to explore the views of young people from excluded communities on self-harm online imagery in terms of motives and effects.
- A balanced sample, inclusive of males and females, offered a wide lens into this topic. Including virtual reality sessions proved to be a positive aspect in enhancing recruitment and engagement.
- The majority of young people were from a refugee and asylum seeker (RAS) background, despite challenging recruitment circumstances related to the COVID-19 pandemic.
- Most participants from the RAS community chose not to use our interpreter, potentially related to the stigma associated with self-harm, which may have limited responses.
- There are some limitations to thematic analysis which are discussed.

## Introduction

 Social media offers a rich source of naturally occurring data^1^ and is increasingly being used in research across disciplines.^2 3^ Initially text based, young people have changed the way they communicate online and are now keen to share their stories, predominantly through imagery, photography and videos.^4^ Young people who self-harm report image-based rather than textual interactions as their primary reason for internet use, and image-based social media platforms such as Instagram have become an increasingly popular and important part of the daily lives of adolescents, partly because they centre around images.^5^ A systematic review, examining the relationship between self-harming behaviours and imagery shared online,^6^ highlighted a number of studies where ethical approval was deemed not necessary to assess social media data. While there is debate on the application of ethical principles in social media research,^7^,^10^ arguably, these are mostly considered from the perspectives of researchers, rather than include the views of young online users.^1^ The idea that young people’s online presence is evidence of apathy towards privacy issues appears misconstrued^11 12^ and there may be little difference between adults and young people in relation to their views on data sharing and privacy protection.^13^

The impact of the online environment and increasing use of imagery on mental health and self-harming behaviours are generally described in positive or negative terms, but this dichotomy is overly simplistic. The impact on individuals may vary—between different individuals but also in the same person at different times.^14^ However, it appears that internet searches for suicide are increasingly returning graphic imagery.^15^ Just over half of young people who self-harm engage in self-harm or suicide-related online searches.^16^ While there are known benefits to online forums through addressing loneliness and isolation, providing support and mitigation of stressful situations,^17 18^ internet use may potentially trigger self-harming behaviours through normalisation and suggestive techniques and practices.^18^,^20^

The rapid change in how young people interact with the online world means it is nearly impossible for researchers to keep abreast.^6^ This may prove particularly challenging in potentially vulnerable young people such as those with lived experience of mental health problems, who self-harm and those from socially excluded groups. Direct engagement with young people is essential to better understand issues relating to the use of their data in a rapidly expanding evidence base and is a key focus of this project.

### Aim

The aim of this study was to explore the views of young people from diverse backgrounds, with or without a history of self-harm, on data sharing specifically in relation to online imagery of self-harm. We explored reasons for sharing self-harm imagery, its potential effects on young people and others and views on sharing/accessing data for mental health/self-harm research.

## Methods

### Theoretical underpinnings

The research was informed by an interpretivist approach that sought to emphasise the social world of participants by documenting their own interpretation of topics related to the research question.^21^ The purpose of this approach is to form a richer understanding of one’s reality in its different contexts, which rejects the guidelines of a positivist led research that promotes a measurable absolute truth that applies to everyone.^22^ This study explored a complex topic that potentially holds a range of socially constructed influences, and therefore required a flexible methodology that allowed participants to voice their personal experiences without having to follow a structured pattern, and in turn, deepened our understanding of their views and perceptions. Following a flexible pattern also allowed for deeper probing into topics that unexpectedly surfaced during the data collection process, and offered richness to the content of the data.^23^

### Sample and recruitment

Participants aged 16–24 years, with or without a history of self-harm and residing in the UK were eligible. Participants were purposively sampled for this study. The definition of self-harm was not predetermined but conceptualised by the participants. Short of the age restriction, maximum variation was sought in order to capture experiences from a wide range of backgrounds. For the first workshop, we aimed to recruit young people from the refugee and asylum seeker (RAS)/sanctuary seeking community. Throughout the recruitment process, as well as in all research materials and information sheets, the term ‘refugee and asylum seekers’ was used, and so is used in this paper to present results from this community. For the second workshop, participants from the general population of the same age group were eligible for inclusion. Recruitment was facilitated by new and existing project partners with local community ties. The principle investigator (AJ) has extensive experience of working alongside people from underserved communities and involving children and young people in self-harm research.^24^,^26^

### Workshops

Workshops were conducted in groups of one to three individuals and were held in two locations one in England and one in Wales over 3 days for each. We aimed to recruit 18 participants for each workshop location. Sample size was determined based on a combination of traditional qualitative research criterion^27^ and richness of data^28^ which we were able to establish following the first workshop. We allowed for non-attendance and last-minute cancellations form participants.

Each participant took part in three activities during their workshop timeslot:

1. Virtual reality (VR) session—a 3D virtual environment to create an abstract representation of their state of mind and allow an opportunity to express their own story through images.
2. PowerPoint presentation related to online imagery for discussion with an artist.
3. Semistructured one-to-one interviews.

Each activity involved data collection and recorded quotes from participants.

### Patient and public involvement

Patient and public involvement was at the heart of this project. This study arose from a pilot project^29^ led by AJ, where young people suggested further research exploring the impact of self-harm imagery in feedback conversations. The semistructured interviews, used in this study, were coproduced with carers, professionals and partner agencies, who have lived experience. Their involvement in the process included design of topics, wording of questions and workshop activities.

### Data collection

Twenty-four interviews were conducted individually, and one interview was conducted with a group of three participants, at their request. Each interview took approximately 45–60 min.

Interview questions were open ended covering the following topic areas: history of self-harm and mental health issues; sharing self-harm imagery online; potential effects of self-harm online imagery on others; data sharing for mental health and self-harm related research.

Participants were provided with information sheets outlining the background and aims of the study, prior to and on the day of the workshops. Study participants were not familiar with the practice of their data being used for research purposes. As such, prior to exploring their views, the interviewer offered a brief outline on how they generate data in their everyday lives, through social media interactions, shopping online, visiting a general practitioner or their grades being recorded in school. We then proceeded to ask them related questions to that topic. Participants had time to read the materials and ask questions followed by them giving informed consent. Participants who were from a RAS background were offered an interpreter during the interviews if required. In line with our ethical approvals and to ensure the safety of potentially vulnerable participants, the interviews were conducted within a setting where ongoing support was available. Interviews were conducted individually with a member of the research team (DD or AJ). In addition, a clinician was on site at all times and available to support participants if this was required. Interviews took place in a designated private room to ensure confidentiality. Interviews were audio recorded, with prior consent from participants and were transcribed verbatim. The interviewers (DD and AJ) had no previous relationship with participants and met with each of them alone or in the presence of an interpreter. The first workshop took place in Wales in June 2021, and the following one took place in England in August 2021.

### Data analysis

Data analysis of semistructured interviews was conducted using thematic analysis^30^ incorporating an inductive approach,^31^ allowing themes to be attained in the context of specific objectives. Thematic analysis was selected with the aim of preserving the level of detail in the data collected, as well is for its nature to enable theory-driven analysis.^30^ This topic had the potential to include several views, partially due to the diversity of the sample, as opposed to one blanket theory, and as such required an exploratory approach. Findings are reported in accordance with consolidated criteria for reporting qualitative research (COREQ) guidelines.^32^

Each individual was given a participant number to replace their name and any names or identifiable information present in transcripts were masked. Completed transcripts were reviewed to ensure participant anonymity was retained.

A coding frame was developed by DD, AM, TS and AJ based on previous literature^1133^,^35^ and an inductive generation of codes.^31^ All transcripts were reviewed by two members of the research team (DD, AM). The coding frame was developed through a process of reading and rereading transcripts, inductive generation of codes and repeated discussions (DD, AM, TS and AJ). Through repeated review of the text, it became apparent which themes were eminent and which are better placed as subthemes. This phase led to the clear definition and naming of each theme. Final coding of interviews was completed by DD and was double coded by AM and TS. This framework was further reviewed and discussed with a third expert researcher (AJ). New codes were added, and themes were consolidated.

A comparative approach was constantly used to identify differences and similarities between the RAS and non-RAS group.^36^

Codes were organised and compiled using NVivo V.22.

### Results and interpretation

This study explored themes related to views of young people from diverse backgrounds, with or without a history of self-harm (see table 1), on the motivation and impacts of sharing self-harm imagery online and the use of their social media data for mental health research. Results are presented in two main themes, encompassing a total of five subthemes (see table 2).

**Table 1:** Participants characteristics

Sample characteristics	ParticipantsN (% of total)	History of self-harmN (% by row)
Total	n=27	n=11 (40)
Sex		
Female	n=11 (41)	n=8 (72)
Male	n=16 (59)	n=3 (18)
Age (years)*		
16	n=6 (22)	
17	n=7 (26)	
18–19	n=6 (22)	
20–24	n=8 (30)	
Background		
Refugee and asylum seeker (RAS)	n=16 (59)	n=2 (12)

* Self-harm is not specified by age in order to avoid possible identification of participants.

**Table 2:** Summary of themes and subthemes that arose from the interviews

Overhead theme	Subtheme	Description
Online imagery of self-harm	Motivations for sharing online imagery of self-harm	This subtheme outlines opinions on why people share self-harm images online.
	Potential effects of sharing self-harm images	This subtheme outlines participants’ views on the potential impact of online images and self-harm.
	Censorship of self-harm images	This subtheme outlines views on whether self-harm images should be censored.
Data sharing for mental health and self-harm research	Use of images and posts for research	This theme describes participants’ views on usage of their posts or images that they share on social media platforms, for research purposes, anonymously or otherwise (both in general and specific to self-harm).
	Trust in organisations for data usage	This theme describes participants’ trust levels in private and public organisations for the purpose of using their data for research purposes.

### Study participants

We recruited 28 study participants aged between 16 and 24 years. One participant was excluded from the study since it was established on attendance that they were over 24 years, leaving a total sample of 27.

### History of self-harm

Participants were asked whether they had experience of self-harm, either personally or through someone they knew. The majority of non-RAS participants reported experience with self-harm as well as knowing other people who self-harmed. Some of them simply answered ‘yes’ when asked this question, while others offered further details on the methods used and motivations. When participants with a history of self-harm were asked further about whether they self-harmed with an intention to end their life, most confirmed that there were incidents where that was their intention: (see online supplemental file 1 for additional self-harm experiences).

Oh yeah definitely. Not the sort of self-harm that everyone thinks of. Not like cutting or things like that but for a little bit I thought I had Dermatol? Anaemia… because I would obsessively like… my cheeks are full of scars, they’re little ones so you can’t see them as much but I would obsessively… anytime I had anything on my face I would just scratch because of my anxiety and I wouldn’t realise that I was doing it. (Female age 20–24)

I’ve been in hospital a few times because of it. It was definitely difficult during the lockdown because not many people were doing face to face sessions for stuff and it’s kind of went for me doing lots of stuff and seeing loads of mates and going to school to being stuck inside all the time (Male age 16–19)

In contrast, just two participants from the RAS group confirmed self-harm behaviours but they did not want to elaborate further. The rest of the participants from this community reported never engaging in any self-harm behaviours nor of knowing anyone who did. Participants from the RAS group often appeared to be worried of being associated with self-harm behaviours. If they did know of someone who self-harmed, it would normally be someone they heard about secondhand rather than someone they knew personally.

A summary of participants’ characteristics is presented in table 1.

### Thematic analysis

The main two overarching themes generated from the data were: *online imagery of self-harm*; and *data sharing for mental health and self-harm research*. Each theme includes anonymised quotes from participants. When applicable within the themes, we highlight differences in responses between the RAS group and the rest of the participants (see table 2 for summary of themes and subthemes).

#### Theme 1: online imagery of self-harm

A primary objective of this study was to explore views on young people’s current online engagement, with a focus on self-harm content, which is currently heavily influenced by imagery. During interviews, we identified three subthemes: one on motives for sharing self-harm images online, the second on the potential effects it may have and the third outlined views on censorship of self-harm images.

Participants acknowledged the importance of having online platforms for sharing content and communicating with populations who were otherwise unattainable. However, the sharing of self-harm images, specifically, was often deemed as a less favourable practice that potentially has the likelihood to carry more negative than positive implications.

#### Motivations for sharing online imagery of self-harm

All participants from both workshops, including those with a history of self-harm, reported never personally sharing a self-harm image online. Non-RAS participants were familiar with self-harm images shared by others and regarded it to be common practice. In contrast, only three RAS participants reported having seen self-harm images online.

Potential reasons for sharing self-harm images were discussed. Responses can be broadly divided into attention seeking, seeking help/connection, raising awareness and expressing pain/creative expression.

The concept that online users who share self-harm imagery content intend to provoke a reaction was expressed. The sharing of self-harm imagery as a form of attention seeking was mostly voiced by participants from a RAS background. Some offered further insight, stating that sharing perhaps stemmed from a need to share their pain and find someone to listen to them. Some participants from the non-RAS workshop also shared the idea of attention seeking.

Attention. 100%, there is no other reason you would put it online. If you wanted it to be a secret, and put it online you are not very clever. It was attention. (Male age 16–19)

I think it depends on the person, if it’s a cry for help about their state or perhaps the self-harming itself is just a bid for attention because I have again known people that have harmed themselves for the culture that comes around it. For the fact that people are asking if you’re OK. For the ability to be to be the victim because for a lot of people that is very helpful (Male age 16–19)

While it appeared overall that most of the sample was somewhat non-sympathetic to users who share images of self-harm online, across both workshops, some suggested that people may be motivated by attention but that this can also be beneficial for raising awareness.

The things that goes through my mind is attention, but I think it’s a good way to raise awareness because it’s happening… people aren’t educated enough on it (Male age 16–19, RAS participant)

Seeking help or connection with others was common feature of discussions:

A lot of reasons. Attention seeking but not in the bad way that more people talk about it… they need something… like it’s not like attention seeking ‘oh he’s such an attention seeker’, it’s like… seeking help… seeking someone to reach out to that will say ‘hey, it’s OK’, seeking people that you can talk to. Relating that there are other people out there and it’s OK, you’re not alone. (Male age 20–24)

I think they want someone to listen to them, but they don’t know who to ask for it. So, they just do their… like the first most legal thing that they can think and it’s just… post an image, to see umm, if anyone would actually care. (Female 16–19, RAS participant)

Maybe like they want to share how they feel with somebody, but nobody is listening to them so maybe they just want to show how are they feeling and what they’re doing and… just… I don’t know. (Female age 16–19, RAS participant)

Maybe they couldn’t talk about it so just go online. (Female age 16–19, RAS participant)

Similarly, some responses specifically addressed what they thought to be a need for creative expression and letting one’s pain out as a release:

It’s another way of getting your pain out, isn’t it? Look I exist… I mean that’s a lot of the reason why people end up doing it… I exist, I am a person, I can feel. And it’s like… sharing a picture… it feels like in the same way that people share art that like… kind of a lot of their feelings are poured into it like that’s why people share it, not to be like, you should do this too. (Female age 20–24)

Possibly to kind of like. I know that lots of people do it with like mental health issues as kind of like… validate their own pain which is bad because everything everyone goes through is valid. (Female age 16–19).

#### Potential effects of sharing self-harm images

The potential impact of viewing self-harm images online was discussed and some of participants’ main concerns related to triggering others who are going through traumatic experiences, and how this content may affect those already vulnerable.

My concern is that they may trigger someone else. Maybe someone’s going through a trauma, or a similar situation and they just don’t want to be reminded of it. They are maybe trying to heal or something. And an image is just bringing them back memories. (Female age 16–19, RAS participant)

Yeah. Umm, I definitely think that it can definitely send people who have issues theirselves… because it kind of brings it to mind. Like if something that’s going on with you… you see it online even if you’ve been able to put it aside, it brings it straight in front of your mind and you can’t get out of it again (Female age 16–19)

Additionally, there was a concern that seeing self-harm images online would normalise self-harm, provide harmful information or promote self-harm as a coping mechanism to young people in distress.

Yeah. Umm, the more they actually look at it, the more they… you see it by itself. The more that this is on social media, the more that people are actually going to do it. That’s absolutely terrifying but it’s true. (Female age 20–24)

One participant explained how exposure to self-harm images online encouraged her to start self-harming. Her views were that surrounding oneself with harmful behaviours, normalises them and enhances the severity of, or adds to existing issues:

I only started self-harming because I knew somebody else who self-harmed… and that sort of exposure to it put that idea in my mind. It’s very like… very much a spreadable thing. I think a lot of the time if you’re around a lot of people who have body image issues or around people who have eating disorders or self-harm or… it can kind of lead you in to developing those problems yourself (Female age 20–24)

#### Censorship of self-harm images

Participants across both workshops expressed the idea that self-harm images should be restricted. Trigger/content warnings were suggested in order to avoid triggering of others or sharing of harmful information (eg, details of methods).

I mean if it’s like you were straight up like putting your self-harm out there then it can be triggering for other people, and it should be like monitored. If you put a photo up and you have scars and things then up … you can’t really remove that because they aren’t focused on it, it’s like… I’m sorry but it’s their type thing. They can put like a trigger warning in place but if it’s not directly them putting out like fresh cuts and stuff online then not really cause, they’re trying to move on from it. (Male age 16–19)

I think warnings for it would be appropriate, I don’t think necessarily removing that sort of thing… if it’s encouraging people to do it then yeah remove it… like… you shouldn’t get temptations like people with eating disorders and stuff… because that’s the real problem that people face (Male age 16–19)

Some suggested that this content should be blocked altogether. It was thought that open access to such content is likely to be presented to individuals who will struggle to cope with it appropriately in effect suggesting that there is no way to determine who can and cannot handle such images:

Just block it… because I don’t want anyone to right, to see images of that so I just want people to block it really because it’s actually really… it’s too sensitive for anyone to see (Female age 16–19, RAS participant)

Others suggested age restrictions over complete removal of content:

I think definitely to under 18s I’d say. I think if you’re an adult you’re kind of hard to filter what you are or aren’t seeing online because you’ve got more access to things. Umm I think in a shock kind of way it can help people to stop if they’re seeing the absolute severity of what can happen. So, each person what they can and can’t handle… (Female age 20–24, RAS participant)

Although agreed by most that self-harm content can be triggering and ought to be restricted, some non-RAS participants expressed an alternate view that sharing of the images serves as a platform of engagement. The importance of having a platform to share distressing experiences and to feel less isolated for people who do not have anyone to turn to, was discussed:

It’s not something I like to see… obviously. But yeah, I think in a way it can be good to have that space to talk about it without that fear of judgement, but I just don’t think that sharing pictures and you know methods is a helpful thing at all. (Female age 20–24)

Maybe images specifically, would be a starting point but if it leads on to sort of general conversations about it like… I could be talking about my experience of depression and that could completely trigger someone else. It could be like, really upsetting for them to read but I also think that it’s so helpful to be able to talk about it so it’s a very difficult topic (Female age 20–24)

### Theme 2: data sharing for mental health and self-harm research

While discussing views on their data being used for research purposes, two subthemes were generated: *use of images and posts for research*; and *trust in organisations for data usage*.

#### Use of images and posts for research

This theme describes participants’ views on use of their posts or images that they share on social media platforms, for research purposes, anonymously or otherwise. We will talk first broadly about the use of posts, and then go on to discuss opinions specific to self-harm related content.

The majority of responses from both groups were positive about sharing their social media posts.

Some participants commented that there is nothing identifiable in posts, whereas others stated that they do not believe this is something they can control anyway, and as such they are mindful of what they share. That said, when it came to using their data, most participants stressed the importance of being able to give their consent:

If it’s information that you’ve publicly posted then it’s acceptable to view it and include it as part of studies or you know big research pooling. It would be… how to control, it would be hard to monitor and manage and it would be easy to be abused so I think it would be better for a consent system to be in place so it would keep people safe as well. (Male age 16–19)

They need to ask first. If they ask, I wouldn’t mind. (Female age 16–19, RAS participant)

I don’t think it’s the worst thing ever, but I think it should definitely be like consent based. Like if that person consents to having their information used then I think you know fine. (Female age 20–24)

Some participants were not happy with the use of images, stating that even if it was for good use, they will likely consider it an unwelcome invasion of privacy, particularly if they are not asked first.

I would really like get angry especially if they didn’t inform me, for like, using them, it’s private. (Male age 16–19, RAS participant)

One participant described the use of images as a possible betrayal of trust. She described how only her social media friends, who she picks carefully, have access to shared images, and as such probably means they shared it without her consent and against her wishes:

Probably not no. I’m quite private on my social media like I don’t have many people and everything’s kind of like… you can only see it if you’re my friends on it. I think the idea of somebody taking my images on social media to use for research… even if it was for beneficial research would seem more like a… like a betrayal of trust because that means somebody who was a friend online has taken those pictures… It’s not just you can stumble upon them quite easily. I don’t think so anyway (Female age 20–24)

A minority of participants were comfortable with the use of their posts or images even without consent. Participants with a history of self-harm highlighted that the purpose of the research was an important consideration in addition to their consent. Participants wanted to make sure that their data were used appropriately, and for a good enough cause:

So, the research topic matters, I think it should be more consensual based on the topic. I would prefer the images I posted weren’t used in political studies because I don’t feel like it’s right to use people’s personal information for political you know… (Male age 16–19)

For research purposes yes but I don’t know, it depends on the context. You could say yes to that and then someone could completely misrepresent how you actually felt, and you may be able to identify yourself from that and be like… (Female age 20–24)

#### Specific to self-harm

Views on using self-harm images for research purposes were divided fairly equally and similarly between the two workshops, into three types of responses; about a third of participants across both groups, who did not have a history of self-harm, were positive about the use self-harm images for research:

Yes, because they are doing that with purpose because they want people to know how they feel. So, I would say yes. (Female age 16–19)

A further third, with a history of self-harm responded negatively stating that it was a breach of privacy and very personal content, which should not be used even for research purposes:

I think this should be private, more private. (Male age 16–19)

I think that’s too much of an invasion of privacy because I think that something like that is very personal to a person and a lot of the time… (Female age 16–19)

Finally, some participants, either with or without a history of self-harm talked about the purpose of the research or, if it is done for the benefit of the public.

Maybe to help like uh to study more about this thing to maybe explore more and maybe produce more ways… alternative ways to like help these people. (Male age 16–19, RAS participant)

The importance of understanding that people self-harm in moments of vulnerability, potentially not worrying about whether, or how their posts be used in future was also discussed.

It depends again on what type of research it is; I can only imagine that kind of research would be beneficial but that’s still someone in a moment of vulnerability. They don’t want it being broadcast even if it is just as a statistic. But at the same time, they posted it online. It’s not like they wanted it to be kept a secret… it’s a very thin… very dangerous line, you step to far on either side and you’ve done something wrong. (Male age 16–19)

#### Trust in organisations for data usage

This theme described participant’s levels of trust towards public and private companies in relation to having access to and using their data or images for research purposes. We asked about the National Health Services (NHS), UK government, universities and private companies such as Google, Facebook or Amazon.

The two most trusted organisations in both groups were the NHS and universities. They were considered to be monitored and regulated organisations where you are able to know how research is being conducted and how data will be handled. Participants also discussed being able to trust information from these organisations:

The NHS would definitely be more trustworthy… because it’s so monitored that… and you know what’s going to happen… (Female age 16–19)

Yeah, I trust the NHS because they give you true information. (Male age 20–24, Ras participant)

There was also a shared agreement by the majority of the overall sample in their lack of trust towards private companies such as Google, Amazon or Facebook when it comes to using their online images for research purposes. There was a common feeling that this was mostly done for profit reasons as opposed to benefiting the public. Participants also expressed that those private organisations, which are typically of a global setting, are not meant to research topics of mental health and self-harm, and should stick to their field:

Places like Facebook are used to share your opinions with friends, it’s not for companies to take your opinions and use them for their own benefit. (Male age 16–19)

I’m not…no, definitely not… I’m not saying they are bad people because they are an organisation, I prefer either to be asked or just say no and once I get into services and get a form then I obviously give my details in that moment if I think I would but… no other ways. (Male age 20–24, RAS participant)

Health organisations can use it to like make statistics and maybe propose some sort of change, but companies definitely wouldn’t… they would just… or even their bots… an algorithm would just try to sell you stuff and try to use that information in other ways so definitely that’s not helpful at all. (Male age 20–24)

When asked about trust levels in the UK government, the RAS group mostly expressed positive feelings. This was not the case for the non-RAS group who expressed a lack of trust in the UK government. One participant explained that the politicians, he is exposed to on tv or in the press did not build trust in the government:

Not with the current information that I have like, if someone was like… this is what is going to happen to it, this is where it goes and this is who deals with it, this is what it’s going to be used for I would. But umm, I think that if I was given the choice, right now, all I know of it being the politicians you see on TV the newspaper articles you read and… I don’t think I would. I would want to know more. (Male age 16–19)

Participants from the RAS group who expressed trust in the government talked about this in comparison to their experiences in their origin country. The UK government was said to have provided support during difficult situations and did not intend them harm. They further felt that the UK government would be there for them should they need it:

UK Government uh yeah I can trust that because if something happened with you, you can go straight away and fix it straight away from a government side if it’s illegal or something… (Male age 20–24, RAS participant)

Yes, I trust government because they try to help us. (Male age 16–19, RAS participant)

Another RAS participant, although expressing trust, also shared her challenges as an asylum seeker in wanting her life to be more private and to be able to put past experiences behind her. She articulated the challenges that come with having to share everything with government:

Yeah. I’m an asylum seeker so… umm… I… we have to tell them everything… to the Home Office and yeah even if… even if we don’t like we have to share that. All about our life back in our countries and here they know everything about us…sometimes you don’t want to talk about the past and they make you talk about the past… (Female age 16–19, RAS participant)

Such reserved responses were in some sense an indicator that the RAS group felt an obligation to express trust in UK authorities. This was either because they felt they were better in comparison to their origin governments, or because they felt they needed to express gratitude for being in this country and as such should not voice any negative views.

## Discussion

This study explored the views of young people from diverse backgrounds, with or without a history of self-harm, on the sharing of data for research specifically in relation to online imagery of self-harm through engaging with young people, exploring motives and possible effects. Although there is published evidence on the effects of engaging with self-harm content, and the influence of images,^537^,^40^ obtaining views from excluded communities, as well as how and if this type of data, that is often freely available online, ought to be used by others, is yet to be explored. We found that young people from all backgrounds care about their privacy and use of their data for research, even when it is publicly available.

Few studies have investigated self-harm among refugees and asylum seekers.^41^,^43^ These typically examine self-harm behaviours by those in immigration detention centres or special community reception centres,^44^ leaving self-harm among young people within this population, particularly during post immigration life, under reported. A recent systematic review,^45^ investigating suicidal ideation and self-harm among young people from migrant communities, identified only five studies up until March 2022, notably not accounting for history of self-harm or suicidal behaviours in their data collection. It was evident that the RAS group was less familiar than other participants with the practice of sharing self-harm images online, as well as more reluctant to discuss this topic on a personal level. Non-RAS participants were also more likely to have a history of self-harm and felt comfortable offering different views and perspectives. The motivations and impacts of self-harm expressed during the interviews were in keeping with existing literature,^46^ suggesting that while exposure to self-harm online offers a digital arena to sharing and exploring, it concurrently may be triggering^47^ and possibly increase self-harm behaviours.^48 49^

The reluctance of the RAS community to engage with questions relating to mental health, likely due to stigma or fear of repercussions, has been highlighted by others.^42 50 51^ Two systematic reviews on suicide and self-harm in young migrants,^44 45^ advised that there was limited literature in this field, and more high-quality evidence was needed.

Participants with a history of self-harm thought people who share their self-harm experiences online might need to feel they are not alone in their distress, get recognition of their pain and some suggested people might share images to raise awareness in a positive way. Young people have previously voiced similar sentiments in relation to attention seeking motivations for sharing self-harm imagery and claimed self-harm ought to be kept private.^5^ This negative attitude to sharing may stem from young people’s belief that it impacts the integrity of the act.^52^ Overall, many in this current sample thought there was a balance to be struck; trying to avoid potentially triggering content, while allowing some provision of a safe environment for those who have no one else with whom to share experiences. The potential impact of self-harm online content has been explored previously,^18^,^20^ but typically from the point of view of those with a history of self-harm or suicidal behaviours. Our sample was not limited to young people with a history of self-harm, since those without a history of self-harm or mental health issues may also view online self-harm content and be impacted by it.

In keeping with existing literature in adults, young people also care about their data privacy,^12 13 53^ even in relation to publicly available social media posts and images. Many expressed that the use of their online posts and images for any research purposes should be with prior consent. While privacy policies are widely used by service providers and social media platforms, terms and condition sections remain vague, long and complicated, with most online users choosing to omit reading them in full, leaving them unaware of what data are being collected and how it is being shared and used.^54^,^56^ Responses to the potential use of self-harm images for research were varied and conflicting but equally distributed indicating similar views between the two groups. Participants were unsure how image data could be anonymised. While most participants wanted to be asked for consent before their own data were used, considering their online data more personal even when publicly available than say health data, they did not express the same level of determination for consent with regard to self-harm images shared by others. Nevertheless, young people who had a history of mental health issues or self-harm were more likely to want explicit consent for the use of their social media data and displayed a higher concern for privacy of sensitive content.

Universities and the NHS were considered the most trustworthy organisations. Participants felt most comfortable with them using their data, feeling confident it would be used for the public’s benefit and not for profit. In contrast, participants expressed a lack of trust in the motives of private companies/industry in using their data. Trust in the NHS and academic institutions has also been supported by other authors,^57 58^ exploring views from adult populations on data sharing.

Responses also differed between the two groups when it came to the UK government, with the RAS group showing more trust particularly when in comparison to their countries of origin. That said, some RAS responses appeared reserved or divergent on this topic, giving the sense it is not for them to criticise anybody of authority in their country of sanctuary. Trust levels and perception in context to the RAS community has been highlighted in literature as being a multifaceted concept influenced by ones background and personal experiences.^59^

### Strengths and limitations

The overall sample of this study was small yet adequate to provide sufficient insight into an under explored topic, particularly among young people from RAS communities. The sample was balanced, inclusive of both male and female which reflected a wider perspective on the topic. Recruitment occurred during the COVID-19 pandemic which was characterised with minimal in-person contact and we adapted workshops to meet COVID-19 restrictions. These challenges were overcome by working with trusted organisations within those communities to facilitate recruitment. The stigma related to the topic itself and using face-to-face interviews for data collection may have also impacted willingness take part and possibly impacted generalisability. Nevertheless, we had no non-attendees for the workshops which was unexpected. Including the VR sessions as part of the workshops, proved to be a positive aspect in enhancing recruitment and engagement, that could be adopted in future studies in underserved young people. Although there was an interpreter present on request for the RAS group, most participants chose to interview without interpreter support, which at times restricted the complexity of responses. This may also reflect their willingness to elaborate on certain topics. Responses given by the RAS group may have been more limited as they felt somewhat less comfortable discussing mental health, suicide and self-harm. These topics are often stigmatised and hidden, but this is potentially increased within their communities.^42^

Thematic analysis does have some limitations, namely that it is less structured than quantitative research, raising more opportunities for the researcher to influence the outcome.^60^ Although inductive coding strives to banish preconceived notions, there is a concern that the researcher’s knowledge and prepossession of ideas can potentially have some level of influence on the themes generated.^61^ That said, a researcher’s subjectivity in qualitative data analysis can also be seen as a valuable resource,^62^ allowing for the natural diversity that exists in thematic analysis putting paid to the idea that a non-positivist approach will likely promote researcher bias.^63^ Braun and Clarke^62^ suggest that the researcher is in fact actively involved in theme construction, and therefore should own their role in how their study is shaped. Having multiple researchers work on the development of the coding frame provides alternative insights and perspectives further enriching this analysis.

### Implications

Coproduction with young people of resources to support understanding and develop innovative solutions to gaining informed consent for data sharing and research for public benefit is required. Young people from excluded communities, postimmigration RAS (since most self-harm studies are in those in detention centres) and males should be purposively involved in future social media research. Themes presented in this paper do not intend to offer a complete account of views on self-harm imagery and data usage. Some important factors have been identified as grounds for future research. This includes the difference in attitudes towards sharing social media data in those with and without a history of self-harm, which requires further investigation. Enhancing the evidence in the online setting in relation to self-harm and suicide will allow for improved prevention measures that can be tailored for excluded communities and their unique characteristics.

### Conclusion

This is the first study to gain views from young people in excluded communities on online self-harm imagery and data usage. This glimpse into the views and understanding of young people in an area, that is already centre stage in their everyday lives, highlighted the fact that although appreciative of the importance of mental health research, and irrespective of background, young people care about their privacy.

## supplementary material

10.1136/bmjopen-2023-076981 online supplemental file 1

## Data Availability

Data are available upon reasonable request.
