# Supplementary material for: #BeSeen: understanding young people’s views of the motivation and impacts of sharing self-harm imagery online and use of their social media data for research—a UK participatory arts-led qualitative study
Source: BMJ Open. 2024 Jul 23;14(7):e076981. doi: 10.1136/bmjopen-2023-076981 (PMC11268025; doi:10.1136/bmjopen-2023-076981)
Supplement: online supplemental file 1 [file bmjopen-14-7-s001.pdf]

Supplementary Material

Participants experiences with self-harm

The majority of non-RAS participants reported experience with self-harm as well as knowing other people who self-harmed:

"Oh yeah definitely. Not the sort of self-harm that everyone thinks of. Not like cutting or things like that but for a little bit I thought I had Dermatol? Anaemia… because I would obsessively like… my cheeks are full of scars, they're little ones so you can't see them as much but I would obsessively… anytime I had anything on my face I would just scratch because of my anxiety and I wouldn't realise that I was doing it." (Female age 20-24)

"I've been in hospital a few times because of it. It was definitely difficult during the lockdown because not many people were doing face to face sessions for stuff and it's kind of went for me doing lots of stuff and seeing loads of mates and going to school to being stuck inside all the time (Male 16-20)

One participant confirmed a history of self-harm while also reporting witnessing her mother do it:

"Both. My Mum actually used to… umm the last time that she did it was… 2017 I think it was, but she didn't know that I was going through so I actually saw her do it." (Female 20-24)

When participants with a history of self-harm were asked further about whether they self-harmed with an intention to end their life, most confirmed that there were incidents where that was their intention. with one of the participants elaborating further on the complexity of her actions:

"it was difficult because I think that's what I had in mind but I also didn't really want to do that. So… you know… the intent wasn't there but I definitely took steps towards it if that makes sense? Like I think truly I didn't want to die when I was doing these things and acting the way that I was but I think it was because I was in such a place of pain that it was the only thing I could think and it was… in a way a kind of comforting thing to think like no matter how bad it gets that option's always there… that's a little bit… a sad way of thinking about it but I think that's a way a lot of people feel." (Female age 20-24)

In contrast, just two participants from the RAS group confirmed self-harm behaviours but they did not want to elaborate further. The rest of the participants from this community reported never engaging in any self-harm behaviours nor of knowing anyone who did. Participants from the RAS group often appeared to be worried of being associated with self-harm behaviours. If they did know of someone who self-harmed it would normally be someone they heard about second-hand rather than someone they knew personally.
Those with a RAS background who did confirm self-harm described it as following:

"I don't really like to say it but yeah I did try… I did try multiple things but pfft, my life decided to say fuck you, you're not dying on me right now. I tried drowning myself, I tried strangling myself, I tried hanging myself, I tried… like… you know high places? I tried jumping off that but none of it worked because my mind just said to me… you have a lot to live for, so yeah… but I don't really like talking about this anyway." Female age 16-20, RAS participant)

"To be honest, umm… I tried myself… umm to hurt myself once and that's why when I see something like this on social media, I try to avoid… watching it. I would rather not say anymore.." (Male age 20-24, RAS participant)
